# The Predictive Value of PAK7 Mutation for Immune Checkpoint Inhibitors Therapy in Non-Small Cell Cancer

**DOI:** 10.3389/fimmu.2022.834142

**Published:** 2022-02-03

**Authors:** Hao Zeng, Fan Tong, Yawen Bin, Ling Peng, Xuan Gao, Xuefeng Xia, Xin Yi, Xiaorong Dong

**Affiliations:** ^1^ Union Hospital, Tongji Medical College, Huazhong University of Science and Technology, Wuhan, China; ^2^ State Key Laboratory of Microbial Resources, Institute of Microbiology, Chinese Academy of Sciences, Beijing, China; ^3^ Research and Development Department, GenePlus- Shenzhen Clinical Laboratory, ShenZhen, China; ^4^ Research and Development Department, Geneplus-Beijing Clinical Laboratory, Beijing, China

**Keywords:** non-small cell lung cancer, immune checkpoint inhibitor, *PAK7*, biomarker, immunity

## Abstract

**Background:**

To date, immunotherapy has improved the 5-year survival rate of patients with advanced non-small cell lung cancer (NSCLC) from 4% to 15%. However, only 30%-50% of the NSCLC patients respond to immune checkpoint inhibitors (ICIs) immunotherapy. Therefore, screening patients for potential benefit with precise biomarkers may be of great value.

**Methods:**

First, an immunotherapy NSCLC cohort was analyzed to identify the gene mutations associated with the prognosis of ICI treatment. Further analyses were conducted using NSCLC cohort in The Cancer Genome Atlas (TCGA) project to validate the correlations between the specific gene mutations and tumor immunogenicity, antitumor immunity, and alterations in the tumor-related pathways using Cell-type Identification By Estimating Relative Subsets Of RNA Transcripts (CIBERSORT) and Gene set enrichment analysis (GSEA).

**Results:**

In the immunotherapy NSCLC cohort (n = 266), significantly longer overall survival (OS) rates were observed in the *PAK7*-mutant type (PAK7-MT) group (n = 13) than the *PAK7*-wild type (PAK7-WT) group (n = 253) (*P* = 0.049, HR = 0.43, 95%CI = 0.23-0.79). In the TCGA cohort, *PAK7* mutations were correlated with the higher tumor mutation burden (TMB) (14.18 *vs.* 7.13, *P <*0.001), increased neoantigen load (NAL) (7.52 *vs.* 4.30, *P <*0.001), lower copy number variation (CNV), and higher mutation rate in the DNA damage response (DDR)-related pathways. In addition, *PAK7* mutations were also positively correlated with immune-related genes expressions and infiltrating CD8+ T cells (0.079 *vs.* 0.054, *P* = 0.005). GSEA results showed that several tumor-related pathways varied in the PAK7-MT group, suggesting the potential mechanisms that regulate the tumor immune-microenvironment.

**Conclusions:**

This study suggested that the *PAK7* mutations might be a potential biomarker to predict the efficacy of immunotherapy for NSCLC patients. Considering the heterogeneity among the patients and other confounding factors, a prospective clinical trial is proposed to further validate the impact of *PAK7* mutation on the immunotherapy outcomes in NSCLC.

## Introduction

Lung cancer has the highest incidence and mortality rates among malignant tumors worldwide, in which non-small cell lung cancer (NSCLC) accounts for 80-85% of lung cancers (1). In recent years, immunotherapy, targeting the immune checkpoints, which include programmed cell death receptor 1 (PD-1), programmed cell death receptor-ligand 1 (PD-L1), and cytotoxic T lymphocyte-associated antigen 4 (CTLA-4), has a great effect on the treatment of NSCLC (2) and has improved the 5-year survival rate of advanced NSCLC from 4% to 15% (3, 4). immune checkpoint inhibitors (ICIs) exhibit durable antitumor effects by activating T cells. However, their response rate in the advanced NSCLC is approximately 30-50% (5–7), which means that quite many patients cannot benefit from this immunotherapy. Therefore, it is crucial to identify novel biomarkers to screen the dominant populations for ICI efficacy.

Fortunately, some biomarkers have successfully predicted the efficacy of ICI treatment to various degrees (8), such as PD-L1 expression, tumor mutation burden (TMB), neoantigen load (NAL), mismatch repair (MMR) status, microsatellite instability (MSI) status, specific gene mutations, and tumor-infiltrating lymphocytes (TILs). Nonetheless, these potential biomarkers still have some limitations. For example, the application of PD-L1 expression is affected by subjectivity in the PD-L1 assays (9), spatial heterogeneity, and temporal variations (10). Furthermore, although the data demonstrating a clinical benefit with better objective response rate (ORR) and progression-free survival (PFS), the association between TMB and overall survival (OS) is not reliable enough (11).. Therefore, the precise predictive biomarkers for ICI treatment are still needed to be explored.

Studies have demonstrated the correlations between specific gene mutations and the efficacy of ICI treatment. Clinical trials have shown the poor efficacy of ICIs for the treatment of NSCLC patients with *EGFR* mutations (12, 13). Patients with co-mutation of TP53 and KRAS altered a group of genes involved in cell-cycle regulation, DNA replication and damage repair, showing remarkable clinical benefit of PD-1 inhibitors (14). Mutations in the STK11 gene in NSCLC patients are associated with an inert tumor immune microenvironment with reduced density of infiltrating CD8+ T cells, thus showing a poor response to ICI therapy (15). These findings suggested that the mutations in tumor-related genes might help in patients’ stratification.

This study aimed to identify the specific gene mutations related to the efficacy of ICI treatment for NSCLC using an NSCLC immunotherapy cohort (16) and The Cancer Genome Atlas (TCGA) NSCLC cohort. The result showed that the *PAK7* mutations were associated with the improved OS of immunotherapy, enhanced tumor immunogenicity, activated antitumor immunity, and alterations in tumor-related pathways, suggesting that *PAK7* mutations might be used as an independent predictive biomarker for the NSCLC patients receiving ICI treatment.

## Materials and Methods

### Clinical Cohorts and Survival Analysis

In order to investigate the correlation between mutations in the *PAK7* gene and ICI efficacy in the NSCLC patients, the Memorial Sloan-Kettering Cancer Center-immunotherapy (MSKCC-IO) cohort, which was a discovery NSCLC immunotherapy cohort (n = 266) was taken from a study by Samstein et al. (16). Then, the immunotherapy cohort was divided into PAK7-MT and PAK7-WT groups according to the nonsynonymous somatic mutation status in the *PAK7* gene and analyzed using Kaplan-Meier survival curves for OS analysis. Furthermore, TCGAbiolinks (17), a R/Bioconductor package, was used to download the somatic mutation and clinical data of the TCGA NSCLC cohort from the Genomic Data Commons (GDC) portal (https://portal.gdc.cancer.gov/). Next, the OS (n = 823) and DFS (disease-free survival) (n = 490) rates of the patients in the *PAK7*-mutant type (PAK7-MT) group in the TCGA cohort were compared with the *PAK7*-wild type (PAK7-WT) group using Kaplan-Meier survival curve analyses. Finally, the correlations between OS and several common driver genes in the MSKCC-IO cohort were determined.

### Genome Characteristics and Tumor Immunogenicity Analyses

The samples in the MSKCC-IO cohort were analyzed using targeted next-generation sequencing (NGS) and evaluated using the Memorial Sloan Kettering Cancer Center-Integrated Mutation Profiling of Actionable Cancer Targets (MSK-IMPACT) test. The NAL data of the TCGA cohort were obtained from the previous study (18). TMB in the TCGA cohort was calculated by dividing the nonsynonymous mutations with 38 Mb as previously reported (19). ComplexHeatmap in the R package was used to visualize the mutational landscape and clinical characteristics of the patients in both cohorts (20). Maftools in the R package was used to visualize the *PAK7* mutation sites and co-mutations in the *PAK7* gene and common driver genes (21).

### Copy Number Variation Analysis

The copy number variation (CNV) data in the TCGA NSCLC cohort were downloaded from the GDC portal using TCGAbiolinks in the R package and using Genome Reference Consortium Human Build 38 (GRCh38) as the reference genome. The data were analyzed with GISTIC2.0 using GenePattern (https://cloud.genepattern.org/gp/pages/index.jsf) platform (22) with default parameter (confidence level was 0.9). The obtained results were visualized using the R package Maftools (21).

### Immune-Related Gene and CIBERSORT Analysis

By Estimating Relative Subsets Of RNA Transcripts (CIBERSORT) (23) (http://cibersort.stanford.edu/) was used to analyze the gene expression data (Illumina HiSeq, RNA-Seq) for comparing the infiltration of 22 immune cells using LM22 signature matrix and 1,000 permutations. The analysis was conducted in the TCGA cohort (n = 887) as well as in an OncoSG cohort (24) (n = 169), which was used as a validation cohort. Besides, the differences in the expression levels of immune-related genes, which were quantified using the log2 (FPKM +1) values obtained from a previous study (18), were also studied along with their functional classification.

### Analyses of the Pathway Enrichment and Mutation Rates in DNA Damage Response Pathways

EdgeR in the R package (25) was used to standardize the raw data of gene expression in the TCGA cohort (n = 887) and conduct differential analysis. Then, clusterProfiler in the R package (26) was used for the gene set enrichment analysis. Four gene sets were obtained using Gene set enrichment analysis (GSEA) from the Molecular Signatures Database (MSigDB) of the Broad Institute (27), which included Reactome, Gene Ontology (GO) terms, Kyoto Encyclopedia of Genes and Genomes (KEGG) pathways, and hallmark gene sets. Pathways with *P*-values <0.05 were considered significantly different. Gene sets involved in the DNA damage response (DDR)–related pathways were obtained from a study by Wang et al. (28). If the nonsynonymous mutations occurred in the genes involved in the DDR-related pathways, the pathway was viewed as mutated in this analysis. Then, the mutation rates in DDR pathways in the PAK7-MT and PAK7-WT groups in the TCGA cohort (n=887) were compared.

### Statistical Analyses

Multivariate Cox regression analysis was performed to identify the prognostic potential of *PAK7* mutations and other common driver gene mutations in the immunotherapy cohort. The correlations of *PAK7* status with TMB, NAL, infiltration level of immune cells, and expression of immune-related genes were evaluated using the Mann-Whitney U test. Fisher’s exact test was performed to assess differences in the mutation status of the top 20 mutated genes and clinical characteristics of patients in both the cohorts between PAK7-MT and PAK7-WT groups. Besides, Fisher’s exact test was also used to analyze the co-mutation status of the *PAK7* gene, common driver genes, and mutation rates in DDR pathways. Survival curves were generated using the Kaplan-Meier method with the log-rank test. A *P*-value of <0.05 was considered significantly different and all the statistical tests were two-sided. R software (version 4.0.3) was used for all the statistical tests and data visualization. The R package ggpurb was used to draw boxplots (29).

## Results

### *PAK7* Mutations Are Associated With a Favorable Prognosis in the NSCLC Patients Receiving ICIs

**Figure 1:**
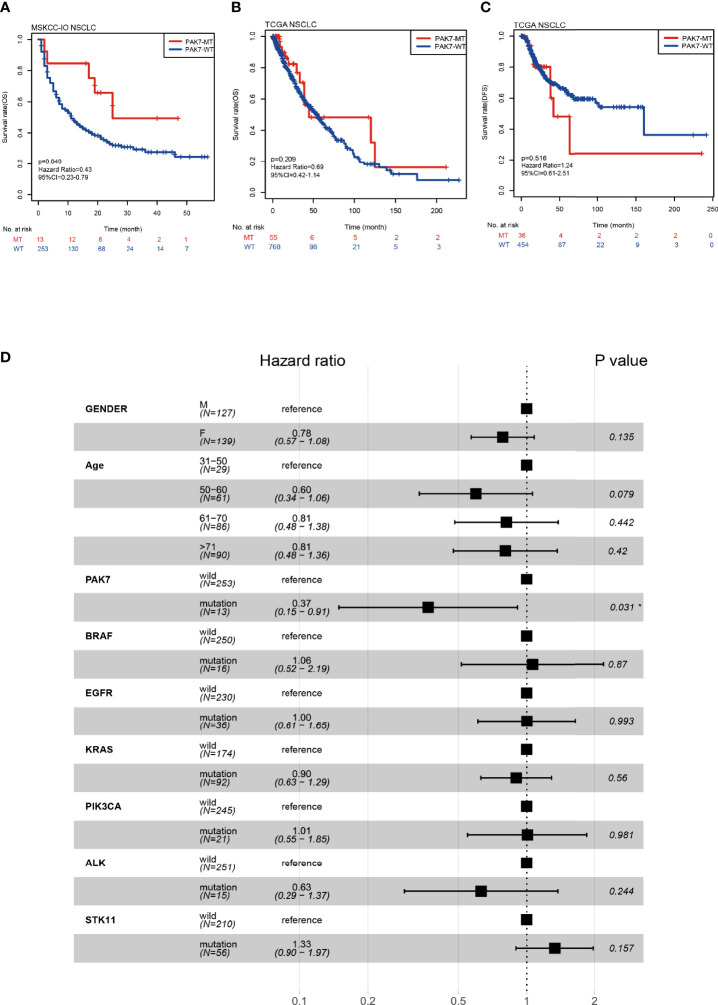
Correlation between PAK7-MT and prognosis of NSCLC patients receiving ICIs. **(A)** Kaplan-Meier analysis of OS for the patients with PAK7-MT or PAK7-WT in the immunotherapy cohort. **(B, C)** Kaplan-Meier analysis of OS and DFS for the patients with PAK7-MT or PAK7-WT in TCGA cohort. **(D)** Forest plot, displaying the results of multivariate Cox proportional-hazard regression analysis of *PAK7* mutation and other common TKI-sensitive gene mutations in the MSKCC-IO cohort. **(A–D)** *P < 0.05.

In early work, we screened all mutations significantly associated with prognosis in immunotherapy cohort (**Supplementary Table 2**), among which *PAK7* mutations were found to mediate enhanced antitumor immunity. The data of an immunotherapy cohort from MSKCC (16) were used as a discovery cohort (NSCLC, n = 266, PAK7-MT *vs* PAK-WT =13:253) in which the patients received inhibitors of PD-1 or PD-L1. The data of TCGA cohort (NSCLC, n = 887, PAK7-MT *vs* PAK-WT =57:830) were also downloaded. Survival analyses were conducted for the MSKCC-IO and TCGA cohorts based on the available clinical and mutation data to investigate the correlations between *PAK7* mutation and clinical outcomes of NSCLC patients. As indicated by Kaplan-Meier analysis, the patients with *PAK7* mutation had significantly better OS in the MSKCC-IO cohort (n = 266, *P* = 0.049, HR = 0.43, 95%CI = 0.23-0.79) (**Figure 1A**). However, no significant differences were observed in the OS (n = 823, *P* = 0.209, HR = 0.69, 95%CI = 0.42-1.14) (**Figure 1B**) or DFS (n = 490, *P* = 0.516, HR = 1.24, 95%CI = 0.61-2.51) (**Figure 1C**) of PAK7-MT and PAK7-WT groups in the TCGA cohort. In addition, multivariate Cox regression analysis was conducted to estimate if the *PAK7* mutation was an independent predictive biomarker. Among the clinical features and other common tyrosine kinase inhibitor (TKI)-sensitive gene mutations (*BRAF*, *EGFR*, *KRAS*, *PIK3CA*, *ALK*, and *STK11*), only *PAK7* mutation correlated with a favorable OS outcome (HR = 0.39, 95%CI = 0.16-0.97, *P* = 0.042) in the MSKCC-IO cohort (**Figure 1D**). Survival analysis for these TKI-sensitive gene mutations (*BRAF*, *EGFR*, *KRAS*, *PIK3CA*, *ALK*, and *STK11*) also showed no significant difference between their mutation and wild-type groups (**Supplementary Figures 1A–F**). In conclusion, the *PAK7* mutations have a considerable potential to predict favorable prognosis independently in the NSCLC immunotherapy.

### Genomic Distinctions Between the PAK7-MT and PAK7-WT Groups

**Figure 2:**
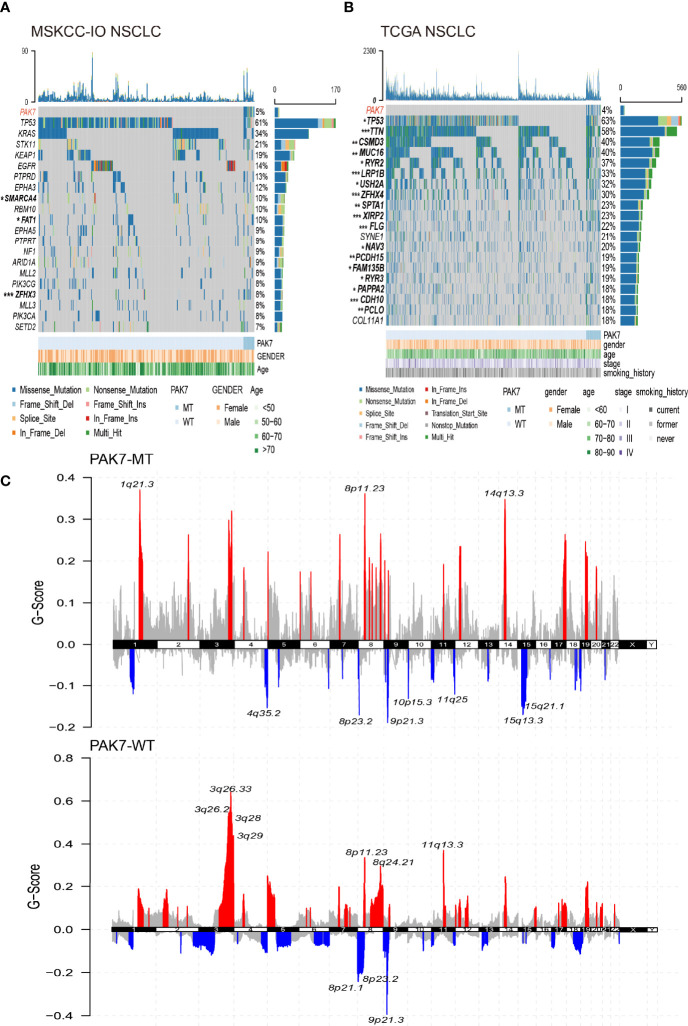
Landscape of somatic mutations and characteristics of *PAK7* mutations in the MSKCC-IO and TCGA cohorts. **(A, B)** Top 20 most frequently mutated genes in the MSKCC-IO and TCGA cohorts. The alteration type, *PAK7* status, and clinical characteristics are annotated. The genes and clinical characteristics with significant differences are highlighted in bold (significance was calculated using Fisher’s exact test). **(C)** Copy number variation in the TCGA cohort. Red and blue colors represent an increase in copy number fragments and a loss of copy number fragments, respectively. The top10 mutation sites have been marked. **(A–C)** *P < 0.05; **P < 0.01; ***P < 0.001.

The genomic distinctions between the PAK7-MT and PAK7-WT groups were investigated. **Figures 2A, B** show the top 20 most frequently mutated genes and clinical characteristics of the patients in the MSKCC-IO and TCGA cohorts. No significant differences were found in the clinical characteristics of patients in both the cohorts, while some of the gene mutation statuses varied between the two groups. In the MSKCC-IO cohort, *SMARCA4* and *ZFHX3* genes mutated more frequently in the PAK7-MT group and the mutation rates of 18 of the top 20 most frequently mutated genes (*TP53, TTN, CSMD3, MUC16, RYR2, LRP1B, USH2A, ZFHX4, SPTA1, XIRP2, FLG, NAV3, PCDH15, FAM135B, RYR3, PAPPA2, CDH10*, and *PCLO*) were significantly higher in the patients with *PAK7* mutation in the TCGA cohort. Among these differential genes, the *TP53, ZFHX3, MUC16, TTN, RYR2*, and *LRP18* gene mutations were reported to be correlated with the enhanced antitumor immunity and favorable prognosis in the immunotherapy of various types of cancers (30–34), which supported the prognostic potential of *PAK7* mutation in the ICI-treated patients. Moreover, the lollipop plots were used to annotate every single *PAK7* mutation in the MSKCC-IO and TCGA cohorts (**Supplementary Figure 2A**). The data from both cohorts showed that the distribution of *PAK7* mutation sites was more even and only p.Glu613Ter mutated twice in the TCGA cohort.

Furthermore, the CNV data in the TCGA cohort was also analyzed after grouping according to the mutation status of *PAK7*. As shown in **Figure 2C**, in the PAK7-MT group, the amplified regions were mainly located on chromosomes 1, 8, and 14 and the deleted regions were mostly located on chromosomes 4, 8 to 11, and 15. However, the PAK7-WT group showed significant variations on chromosomes 3, 8, and 11 and deletions on chromosomes 8 and 9. The bubble plots demonstrate the gene number, sample size, and significance level of the variations in different regions (**Supplementary Figure 2B**). The distribution and G-score of amplified and deleted regions in the PAK7-MT group were significantly lower than those in the PAK7-WT group, which was consistent with the previous studies (35, 36).

It has been a consensus that the NSCLC patients with driver mutation benefit less from the ICI treatment (37) due to low TMB and suppressive antitumor immune microenvironment (38, 39). Therefore, the co-existence of *PAK7* mutation with common driver mutations was investigated. As shown in **Supplementary Figures 3A, B**, no driver gene mutation was co-existed with *PAK7* mutation except the *DDR2* mutation in the MSKCC-IO cohort and *FGFR1* mutation in the TCGA cohort.

### *PAK7* Mutations Are Correlated With Enhanced Tumor Immunogenicity and Alterations in DDR Pathways

**Figure 3:**
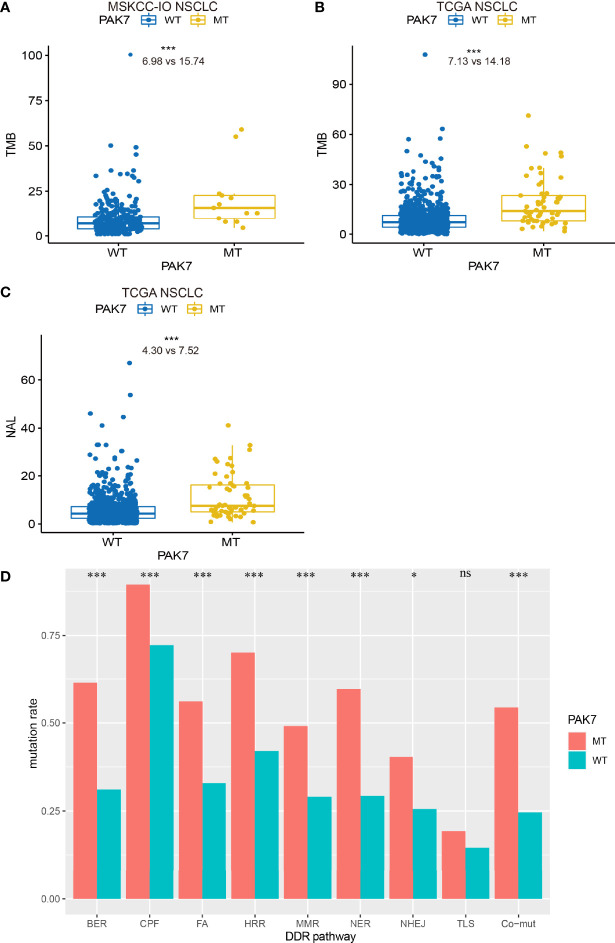
PAK7-MT group correlated with TMB, NAL, and alterations in DDR pathways. **(A–C)** Comparison of TMB (number of mutations per Mb) and NAL (number of neoantigen per Mb) between the PAK7-MT and PAK7-WT group tumors in the MSKCC-IO and TCGA cohorts (Mann–Whitney U test). **(D)** Comparison of mutation rate in the DDR-related pathways between the PAK7-MT and PAK7-WT groups in the TCGA cohort. Co-mutation means the patients with mutations in HRR and MMR (HRR-MMR) or HRR and BER (HRR-BER) (Fisher’s exact test). **(A–D)** *P < 0.05; ***P < 0.001; ns, no significance.

TMB and NAL represent tumor immunogenicity to some degree and are reported to be related to the clinical efficacy of immunotherapy. Therefore, differences in the TMB and NAL between the PAK7-MT and PAK7-WT groups were investigated. As expected, the PAK7-MT group had a significantly higher TMB than that of the PAK7-WT group in both the MSKCC-IO (15.74 *vs.* 6.98, *P <*0.001) and TCGA (14.18 *vs.* 7.13, *P <*0.001) cohorts (**Figures 3A, B**). The NAL of the PAK7-MT group was also significantly higher than that of the PAK7-WT group in the TCGA cohort (7.52 *vs.* 4.30, *P <*0.001) (**Figure 3C**), indicating the enhanced tumor immunogenicity.

Recent studies have indicated that the mutations in DDR pathways, which are quite frequent in NSCLC, are associated with genomic instability and might lead to improved clinical outcomes in the NSCLC patients treated with ICIs (28, 40, 41). Therefore, a total of 8 DDR pathways (**Supplementary Table 1**) were investigated to compare the differences between PAK7-MT and PAK7-WT groups. In the TCGA cohort, the mutation rates of 7 DDR pathways were higher in the PAK7-MT group, which included base excision repair (BER), checkpoint factor (CPF), Fanconi anemia (FA), homologous recombination repair (HRR), mismatch repair (MMR), nucleotide excision repair (NER), and nonhomologous end-joining (NHEJ) (**Figure 3D**). According to Wang et al. (28), the co-mutations of HRR and MMR (HRR-MMR) or HRR and BER (HRR-BER) are associated with the higher TMB, NAL, and immune-regulatory gene expression and predict the favorable outcomes for ICI treatment. Not surprisingly, the occurrence of these two co-mutations was also significantly higher in the PAK7-MT group than that in the PAK7-WT group.

Collectively, these data suggested that the higher TMB, NAL, and mutation rate in DDR pathways in the patients with PAK7 mutation might be related to their better response to ICI immunotherapy.

### *PAK7* Mutations Activate the Antitumor Immunity

**Figure 4:**
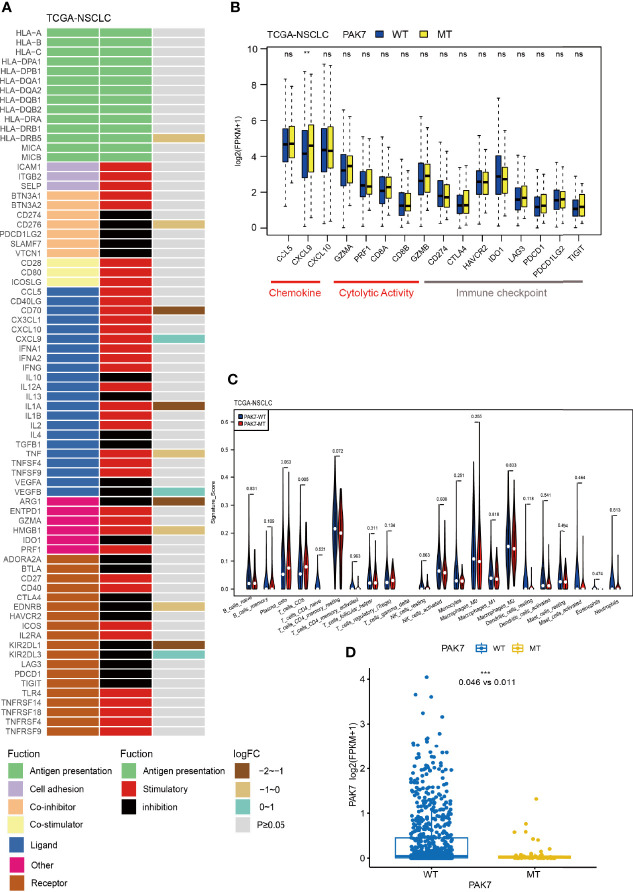
PAK7 mutations are associated with activated antitumor immunity. **(A)** Mean differences in the mRNA expression levels of immune-related genes between the PAK7-MT and PAK7-WT groups in the TCGA cohort. From left to right, each row indicates a gene name and function, immune signature, and log2 transformed fold change (FC, fold change in the mean immune signature enrichment level or ratio). **(B)** Comparison of the expression levels of immune-related genes, such as chemokines, cytolytic activity-associated genes, and immune checkpoints, between the PAK7-MT and PAK7-WT groups in the TCGA cohort (Mann–Whitney U test). **(C)** Infiltration frequencies of 22 types of immune cells in the PAK7-MT and PAK7-WT groups of the TCGA cohort. Signature score stands for the proportion of immune cells. **(D)** Comparison of the expression levels of *PAK7* between the PAK7-MT and PAK7-WT groups (Mann–Whitney U test). **(A–D)**, **P < 0.01; ****P < 0.001; ns, no significance.

The efficacy of immunotherapy depends not only on the immunogenicity of the tumor itself but also on the immune status of the tumor. In order to explore the alterations in antitumor immunity, the relative expressions of 74 immune-related genes in the TCGA cohort in PAK7-MT and PAK7-WT groups were analyzed. The result showed that the expression levels of *HLA-DRB5, CD276, CD70, IL1A, TNF, ARG1, HMGB1, EDNRB*, and *KIR2DL1* genes were significantly lower in the PAK7-MT group, while those of *CXCL9, VEGFB*, and *KIR2DL3* were significantly higher (**Figure 4A**). Among them, the *CD276* gene, also known as *B7-H3*, is a member of the B7 ligand family and is overexpressed in various types of cancers (42). According to a recent study, CD276 could mediate the immune escape in carcinoma stem cells (43), making it an attractive target for antibody-based immunotherapy. ARG1 (arginase 1) is a biomarker of M2 macrophages and has immunosuppressive and tumorigenic functions (44). The mRNA level of *ARG1* has been demonstrated as an adverse prognostic factor for the OS of head and neck squamous cell carcinoma (HNSCC) patients (45). Furthermore, the relative expressions of another set of 16 immune-related genes (chemokines/cytolytic activity/immune checkpoints) in the TCGA cohort were investigated, and found that the expression level of CXCL9 increased significantly in the PAK7-MT group (**Figure 4B**) as compared to the PAK7-WT group. There was evidence that the CXCL9 axis regulated the migration, differentiation, and activation of immune cells, leading to tumor suppression, thereby playing essential roles in the ICI treatment (46–48). Collectively, these differential gene mutations suggested an enhanced antitumor immunity in patients with *PAK7* mutations.

Studies show that the infiltrating immune cells, especially CD8+ T cells, have an important effect on the prognosis of patients, receiving ICI treatment (49, 50). In order to further investigate the immune environment of tumors with *PAK7* mutations, the CIBERSORT LM22 signature matrix was used to estimate the infiltration of 22 types of immune cells in the TCGA cohort. As shown in **Figure 4C**, the CD8+ T cells were significantly more abundant in the PAK7-MT group than those in the PAK7- WT group (0.079 *vs.* 0.054, *P* = 0.005), indicating an activated antitumor immune microenvironment. OncoSG cohort (24), consisting of east Asian lung adenocarcinomas patients (n = 169) was used to revalidate the CIBERSORT result, which showed significant increase in the CD8+ T cells (0.093 *vs.* 0.059, *P* = 0.033), plasma cells (0.167 *vs.* 0.056, *P* = 0.015), and activated memory CD4+ T cell (0.081 *vs.* 0.037,*P* = 0.019) in PAK7-MT group as compared to the PAK7-WT group (**Supplementary Figure 4**). This was consistent with the foregoing conclusion that the *PAK7* mutation could activate the antitumor immunity.

### *PAK7* Mutations Affect the Tumor-Related Biological Pathways

**Figure 5:**
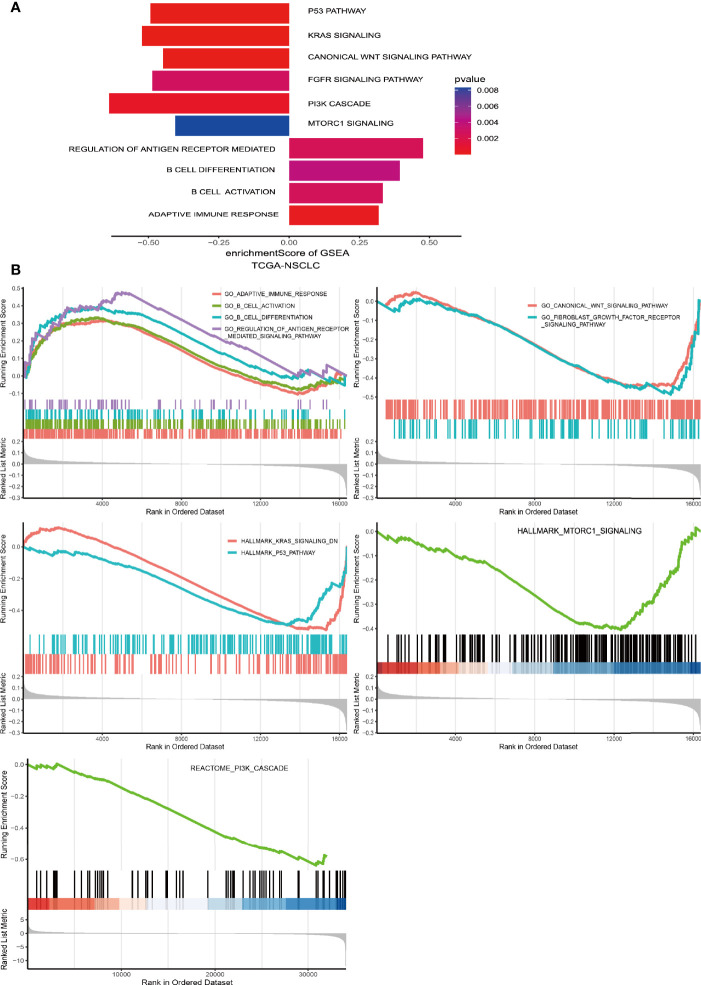
Biological function enrichment analysis of the transcriptomic data of PAK7-MT and PAK7-WT groups in the TCGA cohort. **(A)** Differences in the pathway activities scored by GSEA between the PAK7-MT and PAK7-WT groups in the TCGA cohort. Several important pathways were enriched in the PAK7-MT and PAK7-WT groups with significant correlations. Pathways with positive and negative enrichment scores were upregulated and downregulated, respectively. **(B)** GSEA results of significant immune-related and tumor-related pathways in the PAK7-WT *vs*. PAK7-MT group in the TCGA cohort.

The mechanism of how *PAK7* mutations positively affect the immunogenicity and antitumor immunity is unknown. In order to understand this mechanism, the tumor-related pathways enrichment analysis was carried out and the results were compared in the PAK7-MT and pak7-WT groups in TCGA the cohort. As shown in **Figure 5A**, several immune-related pathways were significantly upregulated in the PAK7-MT group, which included the regulation of antigen receptor-mediated B cell differentiation and activation, and adaptive immune response pathways. In contrast, the oncogenic pathways, such as the P53 pathway, KRAS signaling pathway, canonical WNT pathways, FGFR signaling pathway, PI3K cascade signaling pathway, and mTORC1 signaling pathway, were downregulated in the PAK7-MT group (**Figure 5B**).

## Discussion

In this study, the NSCLC patients with *PAK7* mutations showed a better prognosis than those without *PAK7* mutations in an ICI-treated cohort, independent of the common driver gene mutations, while in the patients, who did not receive immunotherapy, the *PAK7* mutations did not clinically benefit the patients. Then, the changes in tumor immunogenicity were investigated and showed higher TMB, NAL, and mutation rates in the DDR pathways in the PAK7-MT than those in the PAK7-WT group. Besides, some immune-related genes and infiltrating immune cells were found to be significantly upregulated in the patients with *PAK7* mutations. Finally, GSEA analysis was performed to identify several tumor-related pathways to demonstrate the potential mechanism of *PAK7* mutation as a predictive biomarker for the prognosis of NSCLC immunotherapy.

P21-activated kinase 7 (PAK7), also known as PAK5, is an essential member of the PAK Ser/Thr kinase family, which is found to be overexpressed in various types of cancers, including lung cancer, and is considered as an oncogene. As a versatile kinase, PAK7 activates many oncogenic pathways in malignant tumors, such as PI3K/AKT signaling (51–53), SATB1 pathway (54), Egr1/MMP2 pathway (55, 56), GATA1 pathway (57), E47 pathway (58), p65-NFkB/cyclin D1 pathway (59–61), and Raf-1 pathway (62, 63), thereby playing a crucial role in activating the tumor proliferation, invasion, and metastasis and preventing cell apoptosis (53, 64–66). *PAK4*, belonging to the same subgroup as *PAK7* in the PAK family, has also been reported to contribute to the low T cell and dendritic cell (DC) infiltration and a lack of response to PD-1 inhibitors (67). However, the association between *PAK7* and immunotherapy remained unclear.

CNV or aneuploidy is widespread in human cancers. A recent study has indicated that CNV correlates with tumor immune evasion and results in reduced response to ICIs (35). A pan-cancer study conducted by Liu et al. also suggested that the low CNV level showed a favorable response to immunotherapy (36). Researchers have also found that the combination of high TMB and low CNV showed better prediction for the ICI efficacy as compared to their individual predictions either, which might partly explain why the patients with small cell lung cancer (SCLC), a cancer type marked with high TMB and high CNV, showed unfavorable responses to ICIs (68). In the present study, the PAK7-MT group exhibited lower CNVs and higher TMB, suggesting a better prognosis of the patients with PAK7 mutations.

DDR system preserves genomic integrity. Therefore, alterations in this system might lead to genomic instability and higher TMB and NAL (69). Previous studies have shown that alterations in the genes involved in a single DDR pathway might improve responses to ICI immunotherapy (40, 70). Furthermore, defects in one DDR pathway might lead to a stronger dependency on the remaining DDR pathways, thereby suggesting higher genomic instability when there are multiple alterations in different DDR pathways (71). The co-mutations in different DDR pathways, especially the HRR-MMR and HRR-BER pathways, showed better performance in predicting the clinical outcomes of immunotherapy (28). Therefore, the higher mutation and co-mutation rates of DDR pathways might be the reason for higher TMB and NAL in the patients with PAK mutations, which cause a better response to ICI immunotherapy.

Chemokines induce the differentiation and migration of tumor-infiltrating immune cells; therefore, their antitumor effects are worth studying. CXCL9, a ligand of CXCR3, which plays an essential role in the activation and recruitment of CD8+T cells (72), is not only correlated with the improved response of chemotherapy (73) and adoptive cellular therapy (74) but also with the antitumor immune responses following ICI immunotherapy (75), thereby suggesting the contribution of CXCL9 to the activated antitumor immune microenvironment. Thus, the high expression level of CXCL9 might be one of the reasons why the CD8+T cells were enriched in the PAK7-MT group and correlated with better OS in ICI immunotherapy.

Mammalian Target of Rapamycin (mTOR) is a Ser/Thr kinase, which shows activity by its two multiprotein complexes, mTORC1 and mTORC2; mTOR is a central regulator of immune responses (76). mTOR signaling pathway plays an essential role in regulating various immune cells, including neutrophils, mast cells, natural killer cells, γδ T cells, macrophages, dendritic cells, T cells, and B cells (77, 78). It has been a classical view that the inhibition of the mTOR signaling pathway might lead to immunosuppression and the rapalogues (mTORc1 inhibitors) have been used for immune rejection after transplantation (79). However, according to the latest studies, the inhibition of the mTOR signaling pathway can enhance the immune response in some immunological contexts (80). Studies have also shown that the inhibitors of mTORC1 might improve antitumor immunity (81, 82). More importantly, the combination of mTORC1 inhibition and ICI are more effective in tumor control than their individual monotherapies by activating the infiltrating CD8+ T cells (83, 84). Besides, mTORC1 signaling is crucial for regulating the survival, proliferation, and metabolism of cells. It has been reported that the up-regulation of mTOR in malignant tumors facilitates aerobic glycolysis, promotes cell proliferation, and prevents autophagy (76). Up to now, the clinical applications of mTORC1 inhibitors have been well-studied. In the present study, the mTORC1 signaling pathway was significantly downregulated in the patients with *PAK7* mutations and CD8+ T cells were enriched, which were consistent with the results of previous studies.

In order to further investigate the correlation between *PAK7* mutation and the mTORC1 signaling pathway, its upstream regulation was investigated. The immediate upstream regulator of mTORC1 is Ras homolog enriched in brain (RHEB), which is activated by PI3K/AKT signaling pathway (76, 78). GSEA results showed that the PI3K/AKT pathway was also downregulated in the PAK7-MT group, indicating the downregulation of mTORC1 signaling and PI3K/AKT pathway was closely related. Moreover, *PAK7* can regulate the PI3K/AKT signaling pathway by promoting the phosphorylation of PI3K and AKT (51, 52), and the expression level of *PAK7* was found to be significantly lower in the PAK7-MT group than that in the PAK7-WT group (0.011 *vs.* 0.046, *P <*0.001) (**Figure 4D**). Therefore, a hypothesis was proposed that, after the *PAK7* mutation, its expression decreased, which resulted in the downregulation of the PI3K/AKT pathway, leading to the suppression of mTORC1 signaling. The downregulation of mTORC1 activated the infiltration of CD8+ T cells, which led to an enhanced antitumor immune microenvironment. Therefore, the patients with *PAK7* mutations benefited more from the ICI treatment.

The present study had some limitations. First, in the MSKCC-IO cohort, targeted sequencing (MSK-IMPACT panel) was used to detect gene mutations, which might cause selection bias in gene mutations. Second, the application of conclusions in this study might be restricted by the limited number of patients involved. Larger cohorts, especially Asian cohorts, are needed to validate these results. Finally, the frequency of *PAK7* mutations was relatively low in both the MSKCC-IO and TCGA cohorts, which limits the use of *PAK7* mutation alone as predictive biomarkers of immunotherapy.

## Conclusions

In this study, *PAK7* mutation was identified as an independent biomarker for the prognosis of NSCLC immunotherapy. *PAK7* mutations were found to be associated with longer OS, enhanced tumor immunogenicity, and antitumor immunity in an ICI-treated cohort. Furthermore, it was proposed that the PAK7-PI3K/AKT-mTORC1 axis might be the potential mechanism for the predictive effect of *PAK7* mutation. However, further prospective clinical studies and exploration of the molecular mechanism are needed to confirm these results and evaluate the clinical potential of *PAK7* mutation as predictive biomarkers of NSCLC immunotherapy.

## Data Availability Statement

The original contributions presented in the study are included in the article/**Supplementary Material**. Further inquiries can be directed to the corresponding author.

## Ethics Statement

The studies involving human participants were reviewed and approved by Medical ethics committee of Tongji Medical College of Huazhong University of science and technology. Written informed consent for participation was not required for this study in accordance with the national legislation and the institutional requirements.

## Author Contributions

HZ and FT conceived, designed, and wrote the manuscript. YB and LP performed the analysis and interpretation. XG, XX, and XY provided critical comments and suggestions. XD initiated the study and revised the manuscript. All the authors contributed to the article and approved the final version.

## Conflict of Interest

The authors declare that the research was conducted in the absence of any commercial or financial relationships that could be construed as a potential conflict of interest.

## Publisher’s Note

All claims expressed in this article are solely those of the authors and do not necessarily represent those of their affiliated organizations, or those of the publisher, the editors and the reviewers. Any product that may be evaluated in this article, or claim that may be made by its manufacturer, is not guaranteed or endorsed by the publisher.

## Supplementary Material

Supplementary Figure 1
**(A–F)** Kaplan-Meier analysis of OS for the patients with common TKI-sensitive gene mutations in an ICI-treated cohort.

Supplementary Figure 2
**(A)** Lollipop plot shows the distribution of PAK7 mutations in the MSKCC-IO and TCGA cohorts. **(B)** Bubble plots demonstrate the gene number, sample size, and significance level of the variable regions.

Supplementary Figure 3
**(A, B)** Heatmap indicates the co-existence and exclusive correlation between PAK7 mutation and common driver gene mutations in the MSKCC-IO and TCGA cohorts.

Supplementary Figure 4 Infiltration frequencies of 22 types of immune cells in the PAK7-MT and PAK7-WT groups of the OncoSG-LAUD cohort.
