# Supplementary figures and images for: The Predictive Value of PAK7 Mutation for Immune Checkpoint Inhibitors Therapy in Non-Small Cell Cancer
Source: Front Immunol. 2022 Feb 3;13:834142. doi: 10.3389/fimmu.2022.834142 (PMC8886445; doi:10.3389/fimmu.2022.834142)

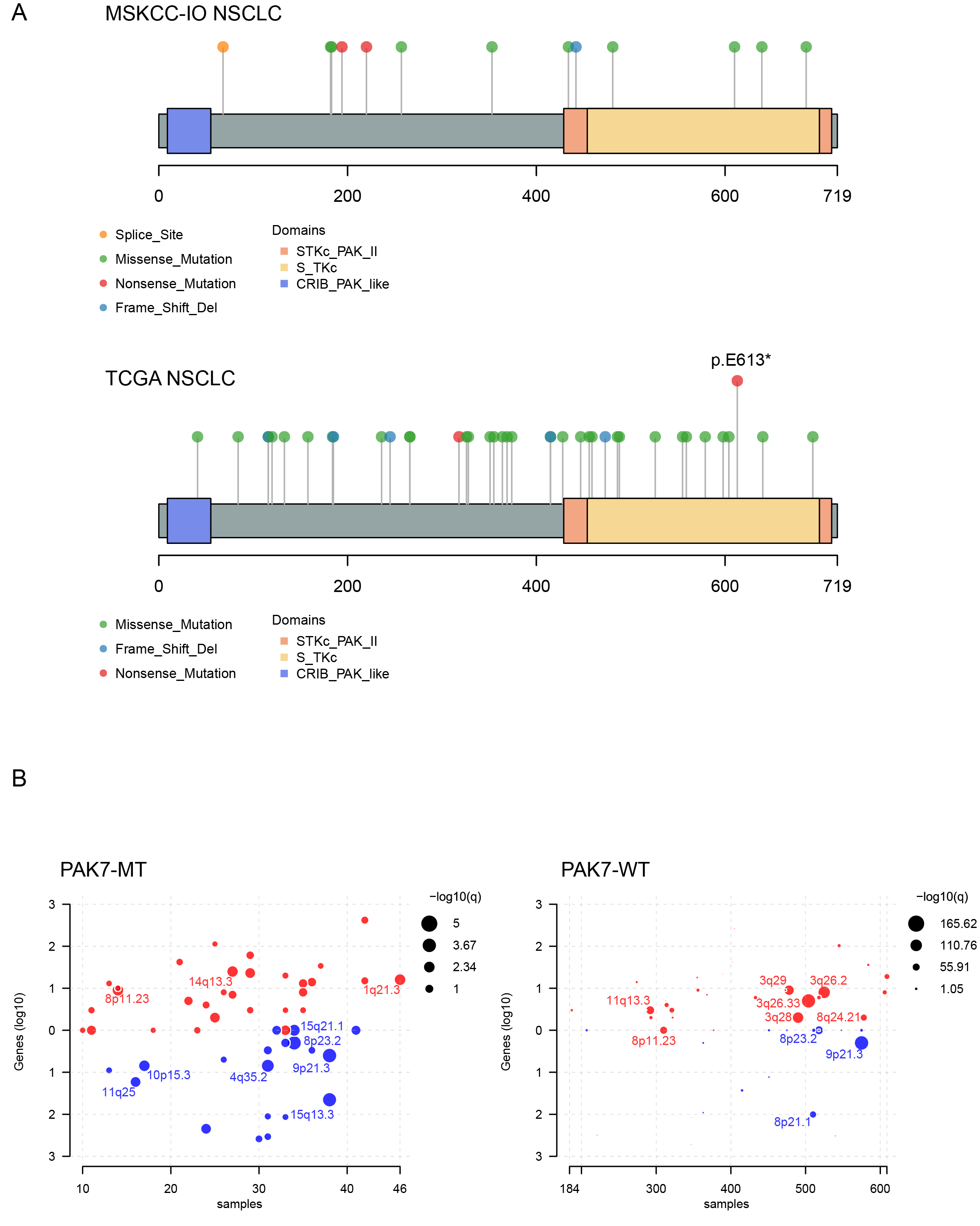

Supplement: Supplementary Figure 1 — (A–F) Kaplan-Meier analysis of OS for the patients with common TKI-sensitive gene mutations in an ICI-treated cohort. [file Image_1.tif]

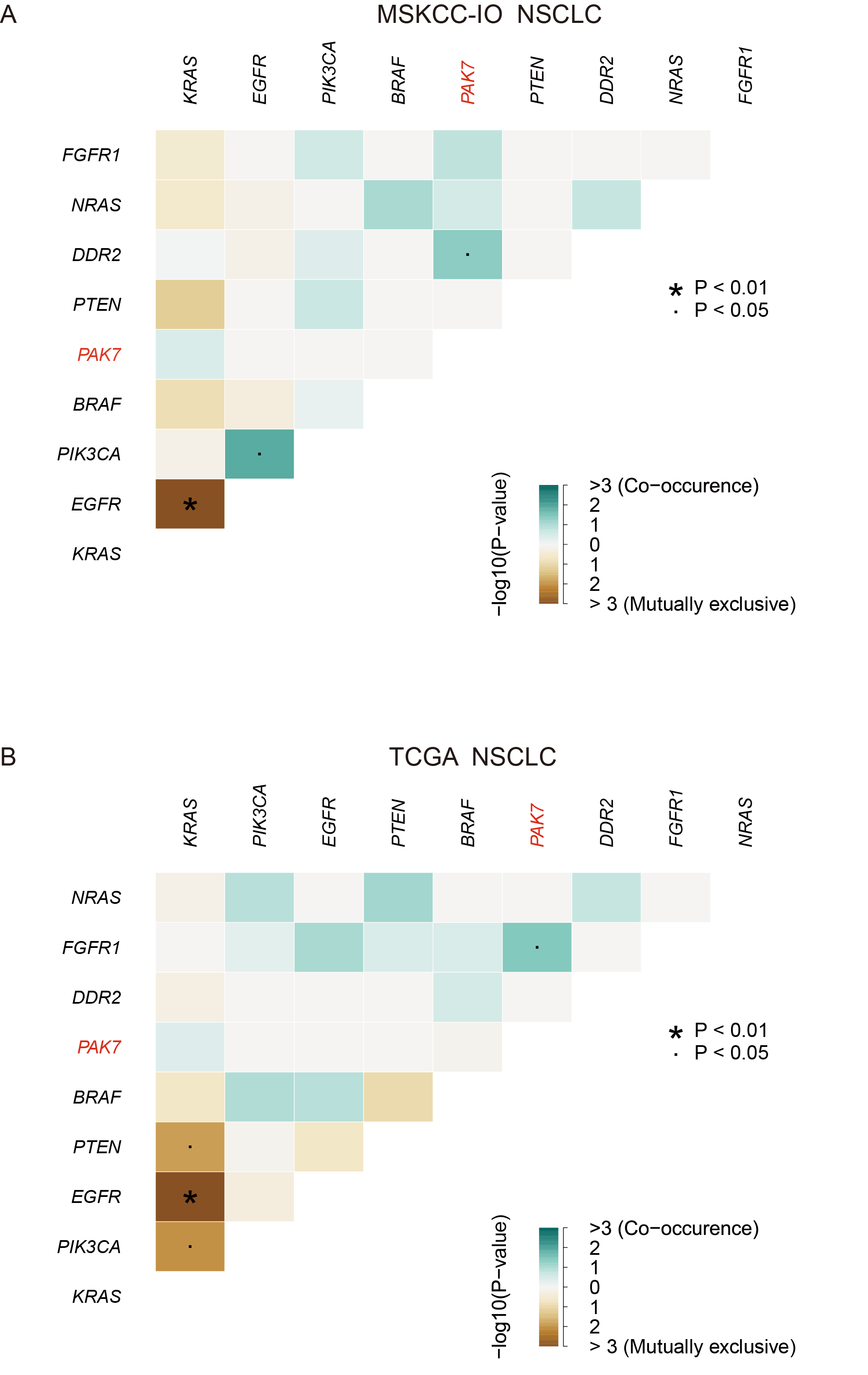

Supplement: Supplementary Figure 2 — (A) Lollipop plot shows the distribution of PAK7 mutations in the MSKCC-IO and TCGA cohorts. (B) Bubble plots demonstrate the gene number, sample size, and significance level of the variable regions. [file Image_2.tif]

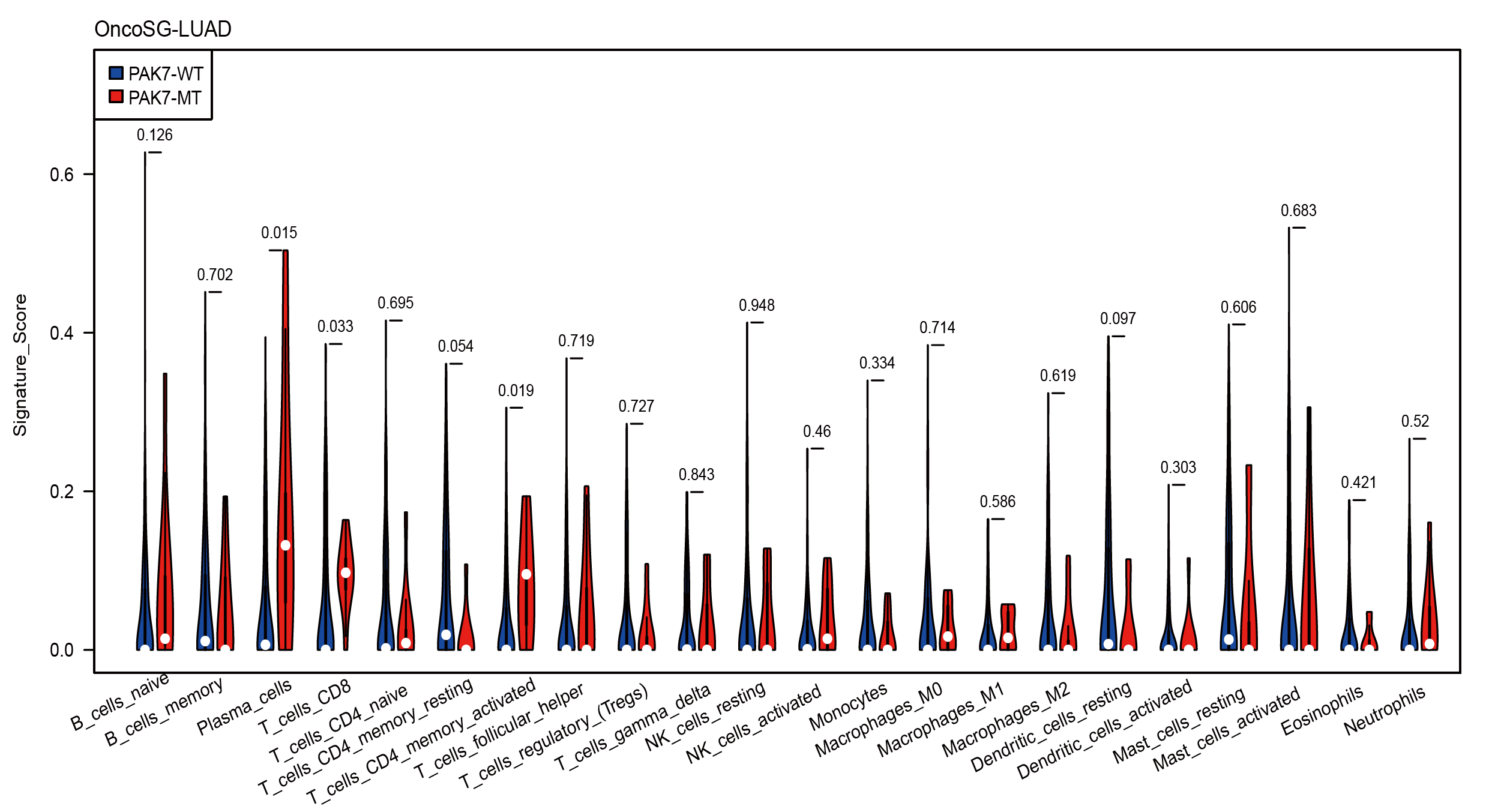

Supplement: Supplementary Figure 3 — (A, B) Heatmap indicates the co-existence and exclusive correlation between PAK7 mutation and common driver gene mutations in the MSKCC-IO and TCGA cohorts. [file Image_3.tif]

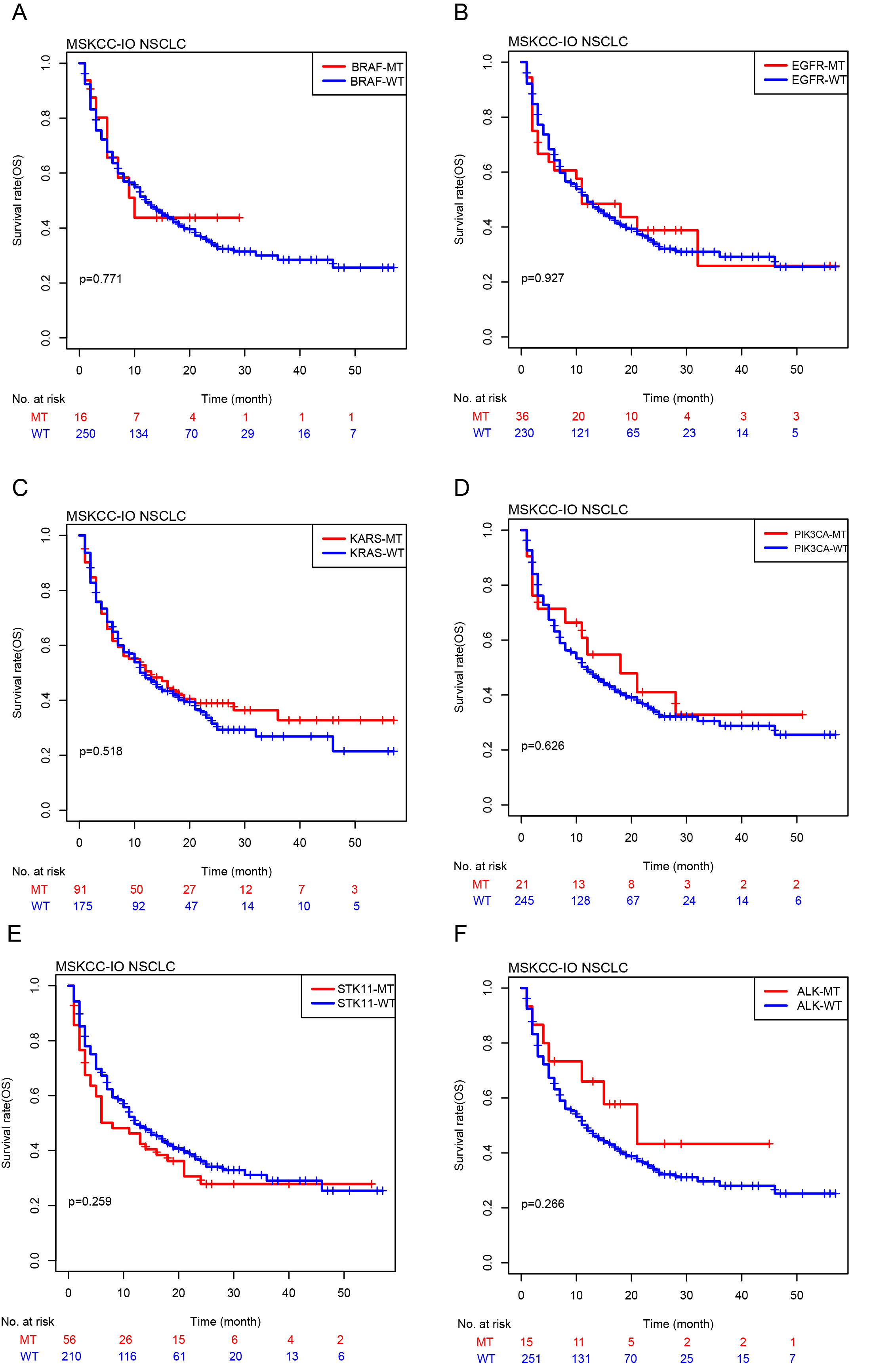

Supplement: Supplementary Figure 4 — Infiltration frequencies of 22 types of immune cells in the PAK7-MT and PAK7-WT groups of the OncoSG-LAUD cohort. [file Image_4.tif]
